# Functional Anterior Knee Pain and Return to Sport Following Bone-Patellar Tendon-Bone Anterior Cruciate Ligament Reconstruction

**DOI:** 10.31486/toj.22.0085

**Published:** 2023

**Authors:** Misty Suri, Arjun Verma, Mohammed Asad Khalid, Michael Nammour, Brian Godshaw

**Affiliations:** ^1^ Ochsner Sports Medicine Institute, Jefferson, LA; ^2^ The University of Queensland Medical School, Ochsner Clinical School, New Orleans, LA

**Keywords:** *Anterior cruciate ligament*, *anterior cruciate ligament injuries*, *anterior cruciate ligament reconstruction*, *bone-patellar tendon-bone grafts*

## Abstract

**Background:** Bone-patellar tendon-bone (BPTB) anterior cruciate ligament (ACL) reconstruction is a frequently used technique but has been associated with a high incidence of postoperative anterior knee pain. However, previous studies have not evaluated if this anterior knee pain is functionally limiting for patients. This study introduces the concept of functional anterior knee pain, or kneecap pain that limits patients’ ability to return to their prior level of activity or sport.

**Methods:** We reviewed BPTB ACL reconstructions from April 2013 to May 2017. Patients included in the analysis had a minimum of 1 year of clinical follow-up and 3 years of survey follow-up. Statistical analyses were performed using paired *t* tests and binomial test.

**Results:** Sixty-seven patients met the inclusion criteria. Compared to the mean preoperative visual analog scale (VAS) pain score of 6.1, patients reported statistically significant reductions in VAS scores at 1 year and 3 years postoperatively to 0.9 and 1.8, respectively (*P*<0.01). The incidence (28.4%) of anterior knee pain was highest at the 3-month time point. This incidence decreased to 6.0% at 1 year and 7.5% at 3 years postoperatively. At 3 years postoperatively, 94% (63/67) of the patients in this study were not limited by functional anterior knee pain and returned to preoperative levels of activity and sport.

**Conclusion:** To our knowledge, this investigation is the first to define and quantify the relationship between postoperative anterior knee pain and resultant functional limitations. This study shows that ACL reconstruction with BPTB autograft was not significantly associated with functional anterior knee pain in our population and that the incidence of postoperative anterior knee pain following BPTB ACL reconstruction may be less than previously reported.

## INTRODUCTION

Arthroscopic anterior cruciate ligament (ACL) reconstruction is a commonly performed procedure, ranking in the top 10 most performed elective orthopedic surgical procedures.^1^ In the United States, more than 200,000 ACL reconstructions are performed annually, costing the health care system approximately $7.6 billion annually.^2,3^ The primary goal of ACL reconstruction is to re-create the anatomy and function of the native ACL so that patients can return to their desired activity level without pain or instability.

Graft selection can lead to various issues postoperatively. The most common autograft choices are quadriceps tendon, quadruple hamstring, and bone-patellar tendon-bone (BPTB). Quadriceps tendon autografts have been associated with significant postoperative weakness in both males and females, with females experiencing significant weakness at 7-year follow-up.^4^ In addition, an investigation by Novaretti et al showed that only 53.4% of patients with quadriceps tendon autografts returned to their preinjury level of sport during a mean follow-up period of 2.1 years.^5^ Quadruple hamstring grafts preserve extensor mechanism strength but have been shown to lead to significant hamstring weakness.^6^ In an investigation of 314 patients with a mean follow-up of 40 months, Ardern et al showed that only 45% of patients receiving autologous hamstring ACL reconstructions were able to return to sport.^7^ A 2020 meta-analysis by DeFazio et al showed that during a minimum follow-up of 1 year, hamstring autografts had an overall return-to-sport rate of 70.6%.^8^ In a 2011 systematic review and meta-analysis that included multiple types of ACL reconstruction grafts, 48 studies, and 5,770 patients, Ardern et al calculated a pooled rate of return to sport of 63%.^9^

To mitigate donor site morbidity, an alternative method is to use allografts. However, allografts also have disadvantages, including increased cost, risk of disease transmission, slower graft incorporation, and a significantly higher failure rate, especially in younger patients (odds ratio 3.87).^10,11^

BPTB autografts may be considered the gold standard for ACL reconstruction. Despite the faster graft incorporation, faster return to sport, and lower re-tear rates or need for revision with BPTB ACL reconstruction, some critics point to postoperative complications as reasons to use other grafts.^10^ In addition to quadricep weakness, patellar fracture is a potentially devastating complication of BPTB autografts.^6,12,13^ Damage to the saphenous nerve resulting in anterior knee numbness has also been reported in up to 53% of patients with BPTB ACL reconstructions.^14^ However, the most commonly cited complication with BPTB grafts is residual anterior knee pain, which has been reported to be as high as 62.9% 3 months postoperatively and is 1.7 times more likely to occur in patients receiving BPTB grafts than in patients receiving quadruple hamstring grafts.^15-17^

Previous reports of anterior knee pain following BPTB ACL reconstruction typically describe this complication as pain with kneeling or with the knee-walk test, in which a patient is asked to walk on the knees and report any difficulty or pain.^12,14,18-22^ However, this type of anterior knee pain is not unique to BPTB ACL reconstruction; in a 2020 study, Calvert et al reported kneeling difficulty in 77% of patients undergoing quadruple hamstring ACL reconstructions.^23^

While the incidence of postoperative knee pain has been widely examined, a paucity of literature has examined the relationship between postoperative anterior knee pain and return-to-sport rates. Because pain while kneeling and the knee-walk test are reflective of a small number of activities of daily living, the results of previous investigations using these tests may not accurately reflect the majority of a patient's postoperative experience. For this investigation, we contend that pain while kneeling and the knee-walk test are not accurate reflections of the functional limitations of postoperative anterior knee pain on patients’ activities of daily living.

We conducted this investigation to specifically explore if postoperative anterior knee pain is a significant causative factor in decreased rates of return to sport following BPTB ACL reconstruction. Therefore, this investigation introduces and delineates a novel explanatory variable, *functional anterior knee pain*, that can be used to assess the postoperative recovery of patients following BPTB ACL reconstruction. Functional anterior knee pain is defined as anterior knee pain that prevents a patient from returning to their prior activity level; it is a variable that other investigations have not specifically identified. Importantly, in introducing the concept of functional anterior knee pain, this investigation provides the novel differentiation of anterior knee pain into functionally limiting and not-limiting subtypes. This differentiation allows investigators to more accurately characterize and evaluate the impact of postoperative anterior knee pain on a patient's capacity for return to prior activity levels and sport.

The primary hypothesis of this investigation was that BPTB ACL reconstruction is not statistically significantly associated with functional anterior knee pain or failure to return to sport. The secondary hypothesis was that no statistically significant difference in return-to-sport rates would be found between BPTB ACL reconstruction and other common ACL reconstruction techniques, such as quadriceps tendon and quadruple hamstring grafts.

## METHODS

This investigation was a single-center, single-surgeon (MS), retrospective review and evaluation of BPTB ACL reconstructions performed between April 2013 and May 2017. Patients included in this investigation had a minimum of 1 year of clinical follow-up and 3 years of survey follow-up. Exclusion criteria included ACL reconstruction with other graft options, revision ACL reconstructions, and <2 years of follow-up. In this investigation, the graft side was ipsilateral to the ACL reconstruction for all patients. For the purposes of this study, functional anterior knee pain was defined as pain on the kneecap that limited patients’ ability to return to their prior activity level.

### Data Collection

After approval from the institutional review board, demographic information—sex, age at time of surgery, and body mass index (BMI)—was collected from the medical records. Information about the surgery was obtained from the operative reports: the laterality of surgery, any additional procedures performed, and dimensions of the harvested BPTB graft and bone blocks. Postoperative clinic follow-up notes were reviewed to obtain visual analog scale (VAS) pain scores, presence of anterior knee pain, and knee range of motion. VAS pain is scored on a scale of 0 to 10, with 0 indicative of no pain and 10 indicative of maximal pain. These notes were available at regular postoperative intervals of 2 weeks, 6 weeks, 3 months, 6 months, and 1 year.

Final follow-up at 3 years postoperatively was conducted via both telephone interviews and electronic surveys. The final follow-up included a survey designed to evaluate satisfaction with the procedure, residual knee pain, and ability to return to sport/activities. Patients who reported the presence of knee pain were asked to specifically identify its location in the knee: kneecap, left, right, or back. With these location options, anterior knee pain was defined as pain reported in the kneecap. To assess the role of this anterior knee pain in causing functional limitations, patients were asked if they had been able to return to prior levels of sport or activities and if the pain was the limiting factor. Additionally, responses to the International Knee Documentation Committee (IKDC) Subjective Knee Evaluation Form, Lysholm Knee Scoring Scale, and Knee Injury and Osteoarthritis Outcome Score (KOOS) were obtained at the final 3-year follow-up. The IKDC evaluation is a patient-reported outcome measure that provides an overall knee function score. The IKDC evaluation is scored on a 0 to 100 scale, with higher scores indicating higher levels of function. The Lysholm Knee Scoring Scale is a patient-reported outcome measure for assessing ACL injuries. The Lysholm scale is 0 to 100, with higher scores indicative of greater levels of functionality and fewer disabilities. The KOOS is a comprehensive assessment of knee injury and knee osteoarthritis. The KOOS is scored on a 0 to 100 scale, with 0 indicative of severe knee problems and 100 indicative of no knee issues.

### Surgical Technique

An examination under general anesthesia was performed to confirm the clinical findings of knee instability. The limb was placed in an arthroscopic leg holder and prepped in standard fashion. Graft harvest was performed first. A longitudinal incision was made over the medial aspect of the patellar tendon. The paratenon was incised longitudinally and preserved for later repair, and blunt dissection was used to expose the distal patella and proximal tibia. The graft was harvested in standard fashion from the central third of the tendon. Once harvested, the graft was taken to the back table and prepared in standard fashion with 2 drill holes for passing sutures in each bone block. A surgical lap sponge was soaked in a solution of 1 g vancomycin powder mixed with 250 cc normal saline and then wrapped around the graft.

The knee was insufflated with saline, and midlateral and inferomedial portals were created. A systematic arthroscopic examination was performed, and any additional pathology was addressed. Meniscus tears were either debrided or repaired using the all-inside technique with Fast-Fix (Smith & Nephew). Attention was then turned to the intercondylar notch. Preparation consisted of minimal notchplasty, and all soft tissue in the over-the-top position was removed with thermal ablation and a curette. The femoral tunnel guide pin was placed into the medial portal at the anatomic insertion site of the anteromedial and posterolateral bundle junction at the 10 o’clock position with the knee at 120° of flexion. After advancement of the guide pin, reaming was done with care to not breach the lateral cortex. The tibial ACL guide was placed at the tibial ACL insertion, and the guide pin was advanced. A core reamer was used, followed by dilators, to create a tibial tunnel the size of the graft. The graft was passed and secured into the femoral tunnel using a metal delta screw (Arthrex, Inc.). After cycling the knee, the tibial side was fixed using a delta screw. Final examination confirmed stable Lachman test.

The patellar tendon was closed with 0 Vicryl suture, and the core reamings were morselized to be used later for autologous grafting of the patellar and tibial harvest sites with maximal fill. The paratenon was closed, followed by the subcutaneous layer and skin.

All patients followed a standardized postoperative rehabilitation protocol.

### Data Analysis

All de-identified patient information was compiled into a secure Microsoft Excel, version 2211 (Microsoft Corporation) spreadsheet. Final follow-up survey and questionnaire responses were recorded using Research Electronic Data Capture (REDCap, Vanderbilt University).^24,25^ Descriptive statistics were used for frequency, mean, median, standard deviation, and normality test. Paired *t* tests were used to compare preoperative and postoperative VAS pain scores. The binomial test was used to compare the return-to-sport rate in this investigation with rates from other published investigations with α=0.05. All statistical analyses were completed using R, version 2022.07.1+554 (The R Foundation).^26^

## RESULTS

**Figure 1.**
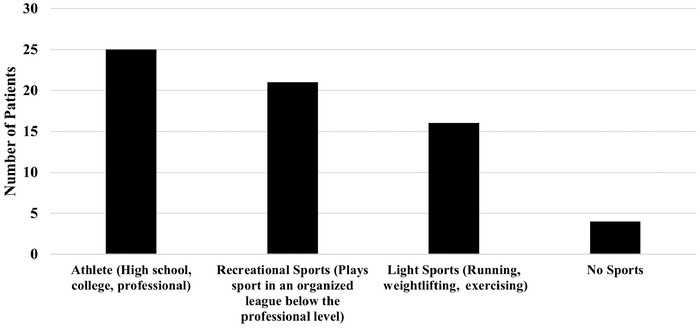
Preoperative activity levels of patients stratified by activity type.

Sixty-seven patients underwent BPTB ACL reconstruction during the study period and met inclusion criteria. Of these 67 patients, 47.8% were male, 52.2% female, and the average age at time of surgery was 29.2 years with an average BMI of 26.6 kg/m^2^ (Table 1). Of these patients, 25 (37.3%) underwent a meniscus repair, 35 patients (52.2%) had isolated partial meniscectomies without repair, and 7 (10.4%) had neither meniscus repair nor meniscectomy. Patients’ preoperative activity levels are shown in Figure 1.

**Table 1.** Patient Demographics and Other Procedures, n=67

Variable	Value
Age, years, mean	29.2
Body mass index, kg/m^2^, mean	26.6
Sex	
Male	32 (47.8)
Female	35 (52.2)
Knee laterality	
Right	32 (47.8)
Left	35 (52.2)
Other procedures	
No meniscus procedure	7 (10.4)
Meniscus repair	25 (37.3)
Medial meniscus repair	18 (26.9)
Lateral meniscus repair	13 (19.4)
Partial meniscectomy (without repair)	35 (52.2)
Medial meniscectomy	21 (31.3)
Lateral meniscectomy	40 (59.7)
Synovectomy	67 (100.0)
Plica excision	63 (94.0)

Notes: Data are presented as n (%) unless otherwise indicated. Several patients underwent surgical repair of both the medial and lateral meniscus.

Table 2 shows mean and range values for tendon graft width, tendon graft length, total graft length, bone block width, femoral bone block length, and tibial bone block length.

**Table 2.** Graft Dimensions

	Dimensions	
Graft	Mean, mm	Range, mm
Tendon graft width	9.9	9-10
Tendon graft length	43.1	30-55
Total graft length	85.6	63-110
Bone block width	10.0	9-10
Femoral bone block length	17.7	10-25
Tibial bone block length	19.1	12-25

**Figure 2.**
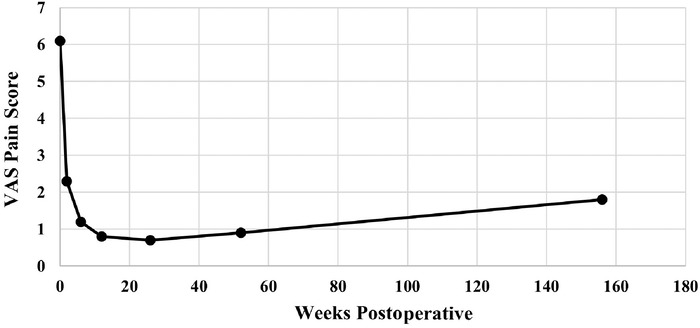
Mean visual analog scale (VAS) pain scores from baseline (preoperatively) to the 3-year postoperative follow-up. The VAS is scored on a scale of 0 to 10, with 0 indicative of no pain and 10 indicative of maximal pain.

**Figure 3.**
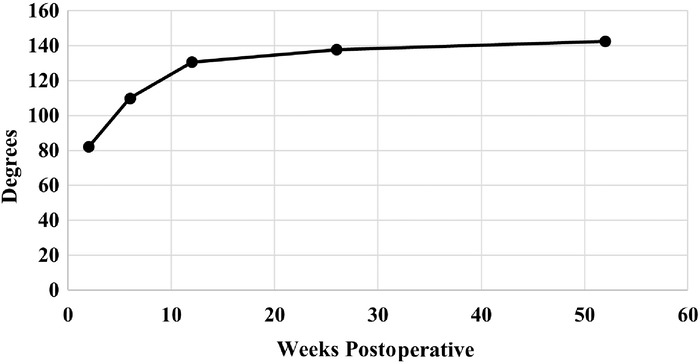
Mean total arc of motion for patients from 2 weeks postoperatively to the 1-year follow-up.

**Figure 4.**
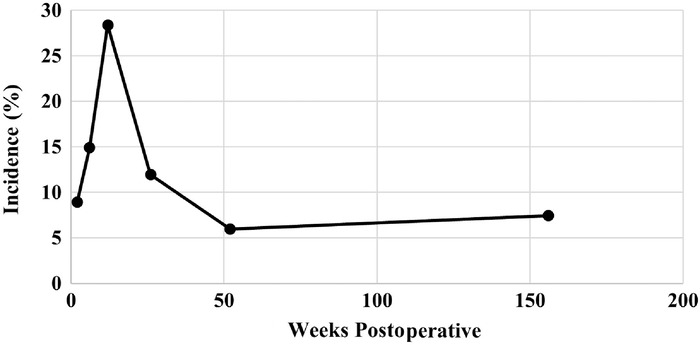
Incidence of anterior knee pain from 2 weeks postoperatively to the 3-year follow-up. The peak incidence (28.4%) occurred at 3-month follow-up.

VAS pain scores decreased during the initial year after surgery, with a mean preoperative score of 6.1 and a mean postoperative score of 2.3 at the 2-week postoperative visit. Mean VAS pain scores decreased to 0.9 and 1.8 at the 1-year and 3-year postoperative time points, respectively, and these reductions were statistically significant (*P*<0.001) (Table 3, Figure 2). At 1 year postoperatively, knee range of motion had increased to a total arc of 142.5° (Table 3, Figure 3). Nineteen patients (28.4%) reported anterior knee pain at their 3-month follow-up (Table 3, Figure 4). The highest incidence of anterior knee pain occurred at the 3-month time point; the percentage of patients reporting anterior knee pain decreased to 6.0% at 1 year and 7.5% at 3 years (Table 3, Figure 4). Median IKDC, Lysholm, and KOOS scores at final follow-up are reported in Table 4.

**Table 3.** Postoperative Outcome Measures, n=67

	Postoperative Follow-Up Time Point					
Outcome Measure	2 Weeks	6 Weeks	3 Months	6 Months	1 Year	3 Years
Visual analog scale pain score, mean	2.3	1.2	0.8	0.7	0.9	1.8
Presence of anterior knee pain, n (%)	6 (9.0)	10 (14.9)	19 (28.4)	8 (11.9)	4 (6.0)	5 (7.5)
Knee range of motion, degrees, mean						
Extension lag	0.8	1.0	0.2	0.6	1.5	
Flexion	82.9	110.8	130.8	138.2	144.0	
Total arc	82.0	109.8	130.5	137.6	142.5	

Note: The visual analog scale is used to score pain on a scale from 0 to 10, with 0 indicative of no pain and 10 indicative of maximal pain.

**Table 4.** Patient-Reported Outcome Scores at 3-Year Follow-Up

Assessment Tool	Score, Median (Q1, Q3)
International Knee Documentation Committee Subjective Knee Evaluation Form^a^	82.8 (72.7, 91.4)
Lysholm Knee Scoring Scale^b^	87.0 (77.0, 93.0)
Knee Injury and Osteoarthritis Outcome Score^c^	
Symptoms	82.1 (75.0, 89.3)
Pain	90.6 (84.4, 96.9)
Activities of daily living	98.5 (94.1, 100)
Sports	85.0 (70.0, 95.0)
Quality of life	75.0 (56.3, 87.5)

^a^The International Knee Documentation Committee Subjective Knee Evaluation Form is a patient-reported outcome measure that provides an overall knee function score. It is scored on a 0 to 100 scale, with higher scores indicating higher levels of function.

^b^The Lysholm Knee Scoring Scale is a patient-reported outcome measure for assessing anterior cruciate ligament injuries. It is scored on a 0 to 100 scale, with higher scores indicative of greater levels of functionality and fewer disabilities.

^c^The Knee Injury and Osteoarthritis Outcome Score is a comprehensive assessment of knee injury and knee osteoarthritis. It is scored on a 0 to 100 scale, with 0 indicative of severe knee problems and 100 indicative of no knee issues.

At 3-year follow-up, 5 patients reported anterior knee pain. Of these 5 patients, 4 patients had functional anterior knee pain that limited their return to activity, and 1 patient was not limited by the anterior knee pain. Therefore, 63/67 (94%) patients were able to return to their previous levels of activity and sport without any functional limitations of anterior knee pain. This return-to-sport rate of 94% for patients undergoing BPTB ACL reconstruction in our population is significantly greater than published rates for both quadriceps autografts and hamstring grafts, as well as the pooled rate of return calculated by Ardern et al^9^ (*P*<0.001) (Table 5).

**Table 5.** Study Variables and Statistical Comparison of Return-to-Sport Rates

	Study			
Variable	Current Investigation	DeFazio et al, 2020^8^	Novaretti et al, 2018^5^	Ardern et al, 2011^9^
Type of ACL autograft	Bone-patellar tendon-bone	Hamstring	Quadriceps	Did not differentiate
Age at surgery, years, mean	29.2	23.1	34.5	25.1
Follow-up, months, mean	36.0	39.4	25.2	41.5
Number of patients	67	1,738	58	5,770
Return-to-sport rate, % (n)	94.0 (63/67)	70.6 (1,033/1,464)	53.4 (33/58)^a^	63.0^b^
*P* value, binomial test		<0.001^c^	<0.001^c^	<0.001^c^

^a^Numbers and percentage are exactly as reported in Novaretti et al.

^b^Ardern et al calculated this percentage as a pooled rate of return from meta-analysis, inclusive of studies with multiple types of anterior cruciate ligament reconstruction grafts.

^c^Statistically significant differences in the return-to-sport rate compared to this investigation, (α=0.05).

One year postoperatively, 27 (40%) patients in this investigation reported discomfort with kneeling. However, of these 27 patients, none reported being limited by this pain.

## DISCUSSION

This investigation shows that reconstruction of the ACL with BPTB autograft in our population resulted in statistically significant differences in rates of return to sport compared to published rates for other reconstruction techniques. At 3-year follow-up, the 94% rate of return to sport and previous levels of activity without any functional limitations of anterior knee pain found in our study indicates that patients may perform better with BPTB autografts compared to published return-to-sport rates for other graft options (Table 5).

Previous studies have reported a high incidence of anterior knee pain after BPTB ACL reconstructions; however, these rates were reported based on the presence of pain while kneeling and/or the knee-walk test.^12,14,18-22^ Evaluating anterior knee pain in this manner likely results in an overreporting of its prevalence. Moreover, the findings of this investigation illustrate that pain while kneeling may not accurately reflect the comprehensive implications of anterior knee pain on activities of daily living or return to preoperative levels of activity. Thus, rather than evaluating anterior knee pain with such tests, we examined whether anterior knee pain limited patients’ ability to return to their previous activity level.

Anterior knee pain after ACL reconstruction has historically been associated with BPTB autografts and has been shown to reach peak prevalence 3 months postoperatively.^15^ In our study, the peak prevalence of anterior knee pain was also 3 months postoperatively (28.4%). Pinczewski et al conducted a prospective cohort trial comparing BPTB vs hamstring autograft ACL reconstructions.^27^ At the conclusion of a 10-year follow-up period, the researchers reported significantly higher rates of both harvest site pain (*P*=0.001) and kneeling pain (*P*=0.01) in the BPTB cohort.^27^ In a meta-analysis of 1,348 BPTB autografts vs 628 hamstring autografts, Freedman et al noted a significantly higher rate of anterior knee pain in patients receiving BPTB autografts (17.4% vs 11.5%, *P*=0.007).^28^ Directly comparing the incidence of anterior knee pain in BPTB vs quadriceps or hamstring autograft ACL reconstructions was outside the scope of our study.

Anterior knee pain is not unique to BPTB ACL reconstructions and is common in hamstring autografts as well. In a prospective randomized controlled trial, Feller and Webster compared BPTB vs hamstring autograft ACL reconstructions and found no difference in anterior knee pain at 3-year follow-up.^29^ In a randomized controlled trial of 64 military patients undergoing BPTB or hamstring ACL reconstruction, Taylor et al reported no difference in anterior knee pain (*P*=0.77) at an average 3-year follow-up.^30^ In an analysis of randomized controlled trials comparing various graft types in ACL reconstruction, Samuelsson et al observed more anterior knee pain in patients with BPTB autografts but noted that the pain was not significant and dissipated with time.^6^ In a 2020 study of hamstring autograft ACL reconstructions only, Calvert et al reported rates of anterior knee pain up to 77% 1 year postoperatively and 54% 2 years postoperatively.^23^ These rates are much higher than the peak incidence in our study of 28.4% at 3 months and the 6% incidence 1 year postoperatively. In a comparison of autografts to allografts, Shelton and Fagan reported the incidence of anterior knee pain to be equivocal between autografts and allografts.^10^

Several theories have been proposed pertaining to the etiology of anterior knee pain following ACL reconstruction.^17^ Damage to the inferior branch of the saphenous nerve is a commonly accepted etiology for anterior knee pain. In a cadaveric study, Kartus et al localized the inferior branch of the saphenous nerve and its relation to BPTB graft harvest and emphasized the importance of protecting this branch during harvest.^31^ One previously accepted cause of anterior knee pain with BPTB graft harvest was the bony defects in the patella and tibial tubercle.^32^ Tsuda et al reported using core reamings to graft both the patellar and tibial harvest sites in 75 patients undergoing BPTB ACL reconstructions.^33^ In their series, Tsuda et al found that a residual patellar bony defect with a depth >2 mm was a statistically significant risk factor for anterior knee pain (9% vs 44%, *P*<0.05). Thus, Tsuda et al illustrated that grafting cored cancellous bone to restore donor site bony defects may decrease the risk of postoperative anterior knee pain.^33^

The technique used in our study incorporated the key takeaways from both Kartus et al^31^ and Tsuda et al^33^ to minimize the risk of developing anterior knee pain. Overall, 94% of the patients in this study had no limitations attributable to functional anterior knee pain at 3-year follow-up.

This study has limitations. First, this investigation was retrospective and included patients from a single surgeon operating at a single center. Next, the way in which the final follow-up data were collected may have led to recall bias in patient responses; however, this type of bias is expected to result in an increased rate of reporting pain or negative outcomes. In their study investigating kneeling pain after knee arthroplasty, Hassaballa et al found that 81% of patients were able to actually kneel when only 37% reported they could kneel.^34^ A more direct assessment of final follow-up anterior knee pain may have yielded different results. Finally, this investigation had a relatively smaller population than the other studies used for comparison.

## CONCLUSION

This investigation introduces the novel explanatory variable of functional anterior knee pain and provides important longitudinal data about postoperative recovery with 3 years of follow-up for BPTB ACL reconstruction. This study demonstrated that ACL reconstruction using BPTB autograft results in a statistically significant increased rate of return to sport compared to the rates published for other techniques. Furthermore, this investigation demonstrated lower rates of anterior knee pain than those reported in the current literature. Overall, 94% of the patients in this study had no limitations attributable to functional anterior knee pain at 3-year postoperative follow-up. The data provided by this investigation indicate that further investigations with longer patient follow-up periods and clearer distinction of anterior knee pain that is functionally limiting are warranted to better elucidate the functionally important variables that determine such outcomes as return to sport after ACL reconstruction.

## Notes

*This article meets the Accreditation Council for Graduate Medical Education and the American Board of Medical Specialties Maintenance of Certification competencies for Patient Care, Medical Knowledge, and Practice-Based Learning and Improvement.*
